# Pre-operative deltoid fatty infiltration as a predictor of outcomes after reverse total shoulder arthroplasty: a systematic review

**DOI:** 10.1016/j.xrrt.2026.100835

**Published:** 2026-08-07

**Authors:** Marcus Allen, Anant Jhaveri, Sarina Zhao, Nicholas H. Maassen, Lewis Shi

**Affiliations:** a The University of Chicago Pritzker School of Medicine, Chicago, IL, USA; b Brown University, Providence, RI, USA; c The University of Chicago, Chicago, IL, USA; d Department of Orthopaedic Surgery and Rehabilitation Medicine, The University of Chicago, Chicago, IL, USA

**Keywords:** Deltoid, Fatty infiltration, Post-operative, Pre-operative, Reverse total shoulder arthroplasty, Goutallier classification, Shoulder outcomes

## Abstract

**Background:**

Reverse total shoulder arthroplasty (rTSA) has become an increasingly common surgery to address various pathologies of the shoulder. The deltoid muscle is the primary driver for shoulder movement following rTSA, and its functionality is integral to rTSA outcomes. The aim of this systematic review was to investigate the impact of pre-operative deltoid fatty infiltration, as seen and quantified through imaging or by the Goutallier classification, on post-operative outcomes following rTSA.

**Methods:**

A systematic review was performed in accordance with the Preferred Reporting Items for Systematic Reviews and Meta-Analyses guidelines. A PubMed and Google Scholar search using the following search terms: “deltoid,” “fatty infiltration,” and “shoulder arthroplasty” was performed. The literature search aimed to identify studies that measured deltoid fatty infiltration and evaluated post-operative outcomes through clinical assessments. One thousand seven hundred sixty-three papers were analyzed and narrowed down for the focus of this review. Inclusion criteria included relevance, quality of research, significance of findings, and number of citations.

**Results:**

Five papers met the inclusion criteria and were included in the review. The results of these papers were summarized. All of these papers were retrospective cohort studies. Higher levels of pre-operative deltoid fatty infiltration, measured by computed tomography, magnetic resonance imaging, or quantified by Goutallier classification, were associated with poorer post-operative rTSA outcomes. This was confirmed through clinical and functional assessments including American Shoulder and Elbow Surgeons, Constant Murley score, Shoulder Arthroplasty Smart score, and Korean Shoulder Scoring System. An association between lower assessment scores and higher levels of deltoid fatty infiltration proved positive in all the studies.

**Conclusion:**

The health of the deltoid muscle is integral to post-operative rTSA outcomes; the amount of fatty infiltration of the deltoid is one potential predictive measure of post-operative clinical outcomes. It is necessary for orthopedic surgeons to be aware of the impact of deltoid fatty infiltration in order to optimize rTSA outcomes for their patients.

Reverse total shoulder arthroplasty (rTSA) was first developed as a solution to rotator cuff incompetent glenohumeral arthritis.^4^ Prior to the rTSA, traditional anatomic total shoulder arthroplasty and hemi-arthroplasty had high failure rates in this population due to the lack of concentric balance normally provided by the intact rotator cuff. rTSA was revolutionary because it obviated the need for rotator cuff stabilization of the joint by moving the center of rotation medially and distally. Reversing the anatomy facilitated this change and created a constrained device that relied heavily on a competent deltoid for stability and function. Since the development of rTSA in the 1980s, its surgical indications have greatly expanded beyond just rotator cuff arthropathy. rTSA is used for a wide array of pathologies including pseudoparalysis with massive cuff tear, complex proximal humeral fractures, malunion and nonunion fracture sequelae, and failed total shoulder arthroplasty.^25^

With the FDA approving rTSA for use in 2003 and the increase in elderly population in the United States, the number of rTSA procedures increased after 2004.^3^^,^^12^ The total number of rTSAs performed is expected to increase in the coming years as projections indicate rTSA may be over 90% of all shoulder replacements by 2040, and the total number of shoulder replacements will grow 700%.^13^ Taking into consideration the increasing importance of rTSA, it is worth reassessing the factors that contribute to success in post-operative outcomes. Considering the deltoid's key role in stability of the prosthesis, elevation, and rotation, identifying factors which might help predict outcomes is important. One area of research has explored the degree of fatty infiltration of muscles involved in the movement, stabilization, and strength of the shoulder.

When muscle fibers atrophy, surrounding fat “infiltrates” the space previously taken by the muscle fibers. Tendon rupture, nerve injury, or neuromuscular disorders may be the cause of muscular degeneration, and the severity of this degeneration may be indicated by several factors. The infiltration of muscles has been documented within atrophied muscles and could be used as a proxy for decreased function, and therefore, a predictor in outcomes for whatever muscle is being assessed. It is an imperfect measure as fatty infiltration does not necessarily mean the muscle will atrophy. Atrophy can also occur without fatty infiltration, but it is information that is likely to be collected with the standard pre-operative tests obtained for presurgical planning. Therefore, this “opportunistic information” could be obtained at no extra cost or time to the patient and might be helpful in predicting outcomes or providing better informed consent.

The amount of fat infiltrating the muscles may be observed through computed tomography (CT) scans as well as magnetic resonance imaging (MRI). The level of fatty infiltration has been previously described, and a classification system was devised for fatty infiltration in the rotator cuff. The Goutallier scale (0, 1, 2, 3, 4) compares the fat to muscle ratio.^8^ Grade 0 means no fatty infiltration, Grade 1 is for tendon thickening and some fatty streaks, Grade 2 is higher levels of fat but less than 50% fat, Grade 3 is equal amounts of fat and muscle, and Grade 4 is more fat than muscle.^8^ The severity of fatty infiltration can also be observed through MRI. In this case, it is graded using a 5-point scale according to the method of Fuchs, which is an adaptation of the 5-point scale described by Goutallier.^27^ The Fuchs scale is an adaptation of the Goutallier scale; however, it is specifically applied to MRI rather than CT and emphasizes fat visibility on MRI sequences.^27^ The current literature predominantly focuses on fatty infiltration of the rotator cuff muscles. Pre-operative fatty infiltration of the rotator cuff is implicated in poor range of motion (ROM) and overall poor outcomes after rTSA.^7^^,^^15^^,^^17^^,^^20^^,^^22^ Specifically, patients with advanced fatty infiltration of the teres minor have worse active external rotation and worse post-operative clinical results—determined using the Constant Murley score.^24^ Studies also demonstrate that supraspinatus and infraspinatus fatty infiltration predicts outcomes in rotator cuff surgery.^7^^,^^22^

Additionally, current literature provides significant information on the impact of muscular fatty infiltration in the knee, hip, neck, and spine.^2^^,^^5^^,^^9^^,^^11^ Jung et al^11^ reported that pre-operative fatty infiltration of the vastus medialis was significantly associated with poorer post-operative functional outcomes after total knee arthroplasty. For unilateral hip arthroplasty, Genc et al^5^ found that the quantity of fat infiltration of the psoas muscle on the implant side greatly affected the amount of muscle atrophy, correlating to poorer clinical outcomes. In the spine, both He et al^9^ and Carvalho et al^2^ have shown that fatty infiltration of cervical paraspinal and erector spinae muscles, respectively, is linked to less favorable clinical outcomes after disc arthroplasty and lumbar discectomy.

Although the rotator cuff contributes to rTSA function, the deltoid is the primary driver of success, and like other large muscles around major joints, its fatty infiltration may influence rTSA outcomes. Further insight into the role of fatty infiltration of the deltoid in abduction, forward elevation, internal/external rotation, and overall strength after rTSA is an area of research that is important. Current literature on fatty infiltration of the deltoid and its impact on rTSA outcomes is limited and fragmented. Therefore, the purpose of this systematic review is to evaluate the existing literature on the connection between fatty infiltration of the deltoid and patient outcomes, aggregate existing evidence, report generalized results, recognize trends, and identify areas that require further research.

## Materials and methods

A systematic review was performed in accordance with the Preferred Reporting Items for Systematic Reviews and Meta-Analyses guidelines.^18^ A comprehensive database search of literature on the relationship between deltoid fatty infiltration and rTSA patient outcome from peer-reviewed journals was conducted on PubMed and Google Scholar. Search terms used included “deltoid,” “fatty infiltration,” and “shoulder arthroplasty.” The initial PubMed and Google Scholar search, covering publications up to April 30, 2025, yielded 1,763 research papers (Fig. 1). Papers were subsequently selected according to relevance, quality of research, significance of their findings, and number of citations. Conference proceedings, pilot studies, and non-English language studies were not eligible for inclusion. Screening and selection were conducted independently by two reviewers and disagreements were resolved through discussion. Upon implementation of exclusion and inclusion criteria, five papers were included in the review (Table I). The modalities used in measuring deltoid fatty infiltration (eg, CT, MRI), ways of deltoid fatty infiltration quantification (eg, Goutallier classification [GC]), associated demographic variables, and clinical tests used for patient post-operative outcomes were identified. Every study included within this review analyzed the effects of pre-operative deltoid fatty infiltration on post-operative outcomes following rTSA. The data from the included studies were grouped according to outcome measures (ex: American Shoulder and Elbow Surgeons [ASES], Constant Murley score (CS), Shoulder Arthroplasty Smart score (SAS), Korean Shoulder Scoring System [KSS], ROM, and strength), and were assessed to identify if common metrics allowed for quantitative synthesis, such as reporting of weighted mean averages. With the expectation of heterogenous outcome measures, a narrative synthesis approach was utilized when statistical methods were not appropriate.

**Figure 1:**
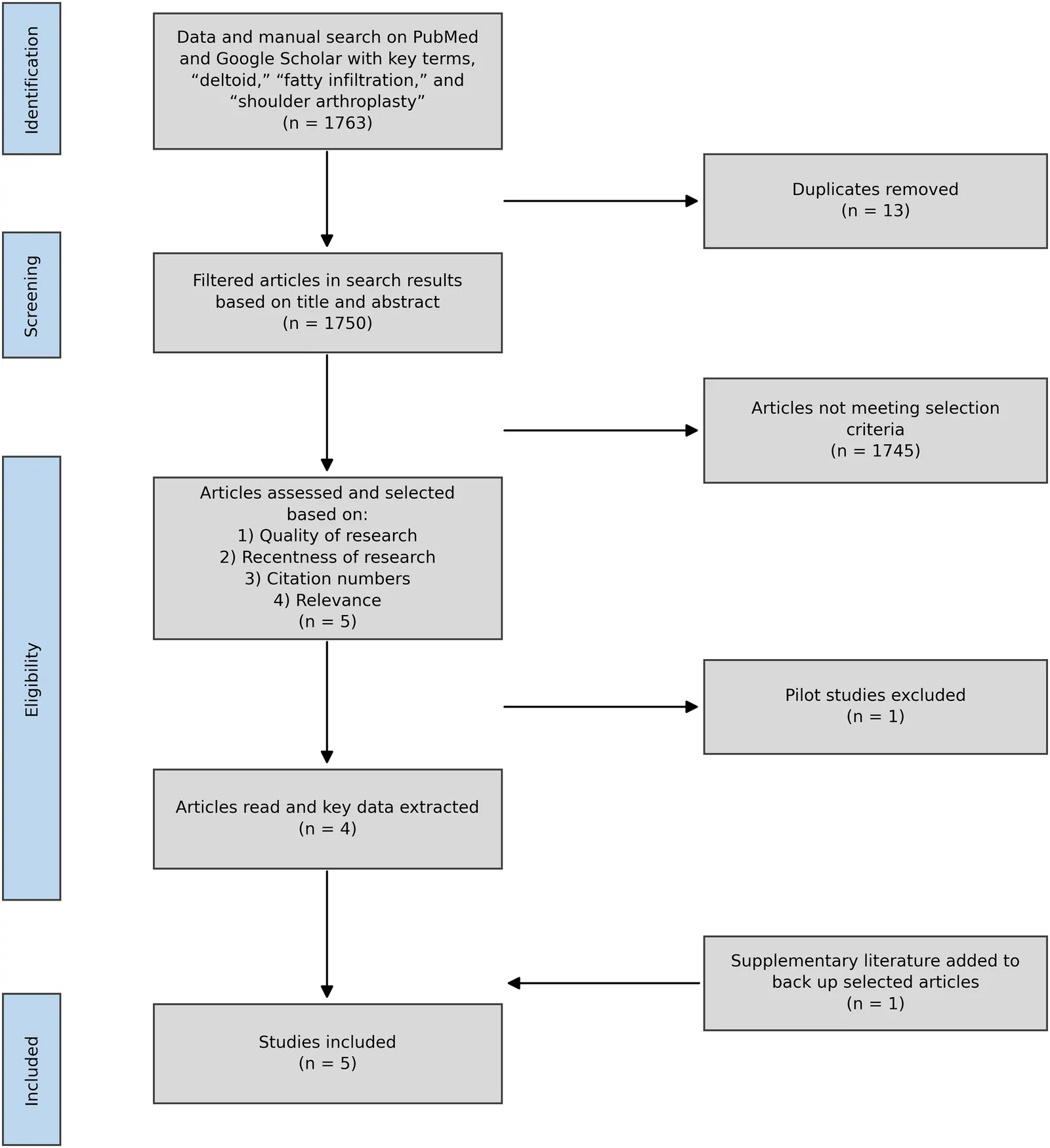
Summary of research methods.

**Table I:** Studies of interest were reviewed and indicated in Table.

Lead author (yr)	Shoulders/patients, n	Mean age, yr	Mean follow-up, mo	Deltoid atrophy measurement/quantification	Shoulder assessment
Lädermann (2013)^14^	49/49	69	38	CT, MRI	CS, ROM
Wiater (2015)^27^	30/30	71.0	30	GC	ASES
Moon (2015)^19^	19/19	71.9	16.1	GC	CS, KSS, muscle strength
Hung (2021)^10^	88/88	67.6	3.6	GC	ASES
Rajabzadeh-Oghaz (2024)^21^	1,057/1,057	70.0	27.1 (aTSA patients), 26.1 (rTSA patients)	BONE kernel CT and FC30 CT	ASES, CS, SAS, ROM, muscle strength

*ROM*, range of motion; *MRI*, magnetic resonance imaging; *CT*, computed tomography; *GC*, Goutallier classification; *aTSA*, anatomic total shoulder arthroplasty; *ASES*, American Shoulder and Elbow Surgeons; *KSS*, Korean Shoulder Scoring System; *CS*, Constant Murley score; *SAS*, Shoulder Arthroplasty Smart score; rTSA, reverse total shoulder arthroplasty.

## Results

Five studies retrospectively examined the impact of pre-operative deltoid muscle impairment and fatty infiltration on outcomes following rTSA. These studies classified deltoid fatty infiltration based on MRI or CT imaging and analyzed its effect on post-operative outcomes through a variety of questionnaires, functional evaluations, and clinical assessments, including ASES score, CS, SAS, KSS, ROM, and strength. Lädermann et al^14^ analyzed 49 rTSA patients with pre-operative deltoid impairment—defined as grade 3 or 4 fatty infiltration—and assessed post-operative outcomes at a mean follow-up of 38 months using ROM, CS, and complication rates. Wiater et al^27^ evaluated 30 rTSA patients with available pre-operative fatty infiltration data and followed them for two years post-operatively, focusing on ASES scores. Moon et al^19^ assessed 19 rTSA patients with pre-operative MRI-based deltoid fatty degeneration, using CS, KSS, and muscle strength as outcome measures. Hung et al^10^ analyzed 88 rTSA shoulders with pre-operative MRI data on fatty infiltration and assessed post-operative ASES scores at a 2-year follow-up. Rajabzadeh-Oghaz et al^21^ employed a machine learning approach to evaluate pre-operative CT images from 1,057 patients who underwent either rTSA or anatomic total shoulder arthroplasty. They investigated how deltoid muscle characteristics, including fatty infiltration, influenced post-operative outcomes such as ASES, CS, ROM, and muscle strength.

### American Shoulder and Elbow Surgeons score

Greater pre-operative deltoid fatty infiltration was associated with significantly worse post-operative ASES scores. Wiater, Rajabzadeh-Oghaz, and Hung utilized ASES as the primary assessment to evaluate post-operative outcomes. Wiater found that quantitatively measured fatty infiltration of the deltoid (7.91% ± 4.32%) had a moderate correlation with decreased post-operative ASES (*P* = .047).^27^ In addition, Rajabzadeh-Oghaz et al^21^ found that patients with greater pre-operative fatty infiltration had significantly lower post-operative ASES scores for both BONE and FC30 kernel cohorts. For the BONE kernel CT image cohort, patients with less than 13% deltoid fat had a 2-year minimum post-operative ASES score of 83.6 ± 17.7, while patients with more than 13% deltoid fat had a 2-year minimum post-operative ASES score of 76.7 ± 19.8.^21^ For the FC30 kernel CT image cohort, patients with less than 28% deltoid fat had a 2-year minimum post-operative ASES score of 83.0 ± 16.0, while patients with more 28% deltoid fat had a 2-year minimum post-operative ASES score of 76.4 ± 20.4.^21^ Along the same lines, Hung determined that ASES functional sub-scores were significantly lower in patients with a deltoid muscle GC of grade 2 (26.1 ± 14.6) than those with grade 0 and grade 1 (38.0 ± 11.6 and 34.8 ± 11, respectively; *P* = .03).^10^

### Constant Murley score

Greater pre-operative deltoid fatty infiltration was associated with worse post-operative Constant scores. Rajabzadeh-Oghaz, Moon, and Lädermann used CS to examine post-operative outcomes. Rajabzadeh-Oghaz et al^21^ performed both CS and ASES. They reported that patients with greater fatty infiltration had significantly lower CS for both the BONE and FC30 kernel cohorts. For the BONE kernel CT image cohort, patients with less than 13% deltoid fat had a 2-year minimum post-operative CS score of 74.0 ± 12.6, while patients with more than 13% deltoid fat had a 2-year minimum post-operative CS score of 61.6 ± 12.6.^21^ For the FC30 kernel CT image cohort, patients with less than 28% deltoid fat had a 2-year minimum post-operative CS score of 73.4 ± 13.1, while patients with more 28% deltoid fat had a 2-year minimum post-operative CS score of 66.6 ± 13.1.^21^ Moon categorized the amount of pre-operative deltoid fatty degeneration using a threshold GC of 1.5. If a patient had a GC higher than 1.5, they were placed in group 2. If a patient had a GC lower than 1.5, they were placed in group 1. Within this study, group 1 had a CS of 75.70 ± 7.39, and group 2 had a CS of 60.00 ± 9.75 (*P* = .002).^19^ Moreover, Lädermann et al^14^ found that the post-operative CS was significantly lower in patients with global impairment (41) compared with partial impairment (57). An important component of deltoid impairment was radiological evidence of grade 3 or 4 fatty infiltration on CT or MRI scans.

### Shoulder Arthroplasty Smart score

Patients with higher pre-operative deltoid fatty infiltration demonstrated significantly lower post-operative SAS scores. Rajabzadeh-Oghaz et al^21^ used SAS to analyze the impact of pre-operative deltoid fatty infiltration on post-operative outcomes. rTSA patients with greater fatty infiltration had significantly lower SAS scores. For the BONE kernel CT image cohort, patients with less than 13% deltoid fat had a 2-year minimum post-operative SAS score of 78.9 ± 10.8, while patients with more than 13% deltoid fat had a 2-year minimum post-operative SAS score of 72.7 ± 11.2.^21^ For the FC30 kernel CT image cohort, patients with less than 28% deltoid fat had a 2-year minimum post-operative SAS score of 76.2 ± 10.1, while patients with more 28% deltoid fat had a 2-year minimum post-operative SAS score of 73.0 ± 10.9.^21^

### Korean Shoulder Scoring System

Moon et al conducted the only study that used KSS to examine post-operative outcome. They reported that group 1, which had less deltoid fatty infiltration, had significantly better clinical outcomes than group 2, which had more fatty deltoid infiltration, based on KSS. KSS was 79.80 ± 4.92 in group 1 and 65.89 ± 9.91 in group 2 (*P* = .002).^19^

### Range of motion

Greater pre-operative deltoid fatty infiltration was associated with significantly worse post-operative ROM—particularly forward elevation and internal rotation—after rTSA. Lädermann and Rajabzadeh-Oghaz used ROM to assess the impact of pre-operative deltoid fatty infiltration on post-operative outcomes. Lädermann demonstrated that, among all patients with deltoid impairment, the mean active forward elevation improved from 50° (sd 38; 0°-150°) pre-operatively to 121° (sd 40; 0°-170°) at final follow-up (*P* < .001).^14^ The mean post-operative forward elevation was significantly lower in patients with global deltoid impairment (70°), compared to those with partial impairment localized to specific regions of the deltoid.^14^ Additionally, the mean active external rotation with the arm at the side increased from 5° (sd 21, −40° to 70°) pre-operatively to 12° (sd 16; −20 to 45°) at the final follow-up (*P* = .844).^14^ Rajabzadeh-Oghaz et al^21^ found that patients with greater fatty infiltration had significantly less forward elevation and internal rotation at a minimum of 2 years after rTSA. For BONE kernel CT images, patients with less than 13% deltoid fat had a 2-year minimum post-operative active forward elevation of 153.7 ± 21.3, while patients with more than 13% deltoid fat had a 2-year minimum post-operative active forward elevation of 140.1 ± 22.3 (*P* < .0001).^21^ For BONE kernel CT images, patients with less than 13% deltoid fat had a 2-year minimum post-operative internal rotation score of 5.0 ± 1.4, and patients with more than 13% deltoid fat had a 2-year minimum post-operative internal rotation score of 3.6 ± 1.9 (*P* < .0001).^21^

### Muscle strength

Higher pre-operative deltoid fatty infiltration was associated with significantly lower post-operative muscle strength. Moon and Rajabzadeh-Oghaz used muscle strength to evaluate post-operative outcomes. Moon illustrated that the post-operative muscle strength of forward elevation was 10.16 ± 2.58 kg (22.40 ± 5.68 lb) in group 1, those with lower fatty degeneration, and 6.68 ± 0.91 kg (14.72 ± 2.00 lb) in group 2, those with higher fatty degeneration (*P* < .001).^19^ The strength of abduction was 9.36 ± 2.67 kg (20.63 ± 5.89 lb) in group 1 and 6.00 ± 0.93 kg (13.22 ± 2.06 lb) in group 2 (*P* < .001).^19^ Rajabzadeh-Oghaz et al^21^ found significant differences in post-operative muscle strength depending on the extent of pre-operative deltoid fatty infiltration. For BONE kernel CT images, patients with less than 13% deltoid fat had a 2-year minimum post-operative strength (max weight) of 10.3 ± 5.0, while patients with more than 13% deltoid fat had a 2-year minimum post-operative strength (max weight) of 7.7 ± 4.0 (*P* = .0006). For the FC30 kernel CT image cohort, patients with less than 28% deltoid fat had a 2-year minimum post-operative strength (max weight) of 9.7 ± 5.6, and patients with more than 28% deltoid fat had a 2-year minimum post-operative strength (max weight) of 5.9 ± 3.7 (*P* < .0001).^21^

### Post-operative complications

Lädermann conducted the only study that examined post-operative complications following rTSA within a patient population with fatty infiltration. Post-operative complications occurred in nine (18%) patients and five (10%) required further surgery.^14^ With that said, there was no difference in the incidence of complications between the different etiologies or the different locations of the deltoid impairment. Some of these complications included dislocations, acute neurological lesions, and infections.^14^ The dislocation rate was 4%.^14^

## Discussion

The present study accumulated existing literature on the effect of pre-operative deltoid fatty infiltration on post-operative outcomes following rTSA. Throughout all the studies analyzed, the common finding was that higher pre-operative levels of deltoid fatty infiltration were correlated with poorer post-operative outcomes following rTSA. More pre-operative deltoid fatty infiltration was associated with worse post-operative shoulder pain, function, strength, and ROM. These correlations existed across the wide variety of measurement tools used to determine post-operative outcomes. Understanding this pre-operative risk factor allows orthopedic surgeons to alter their treatment plans to optimize surgical outcomes as well as better inform and advise their patients about the risks and benefits of surgery.

The findings of this review align with existing research exploring the effects of pre-operative fatty infiltration on post-operative outcomes in different regions of the body. Fatty infiltration of the vastus medialis, psoas muscle, cervical paraspinal muscle, and erector spinae muscles were also correlated with poorer clinical outcomes for total knee arthroplasty, unilateral hip arthroplasty, disc arthroplasty, and lumbar discectomy, respectively.^2^^,^^5^^,^^9^^,^^11^

Although deltoid fatty infiltration leads to poorer post-operative outcomes, there are other risk factors, both socially and physically, that have been associated with worse outcomes following rTSA. While reviewing the literature on fatty infiltration of the deltoid pre-rTSA, results found coincidence of fatty infiltration of the teres minor and hypertrophy of the teres minor to be negative predictors of post-operative outcomes. Patients with less pre-operative fatty infiltration of the teres minor generally demonstrated significantly higher strength with external rotation post-rTSA than patients with more pre-operative fatty infiltration of the teres minor.^19^ There was not a significant difference with forward elevation and abduction depending on the amount of pre-operative teres minor fatty infiltration, but those with less pre-operative teres minor fatty infiltration generally displayed more strength post-operatively.^19^ Aibinder performed a retrospective review of 7,376 consecutive non-contrast shoulder MRI studies for shoulder pain.^1^ He et al^9^ found that concomitant deltoid muscle fatty infiltration occurred in 68% of shoulders with teres minor fatty infiltration. This is not surprising given both muscles are innervated by the axillary nerve. Further clinical studies to assess the quality of the deltoid muscle in patients with teres minor fatty infiltration are necessary to understand their relationship as well as how teres minor fatty infiltration affects post-operative outcomes post-rTSA.

Furthermore, it is well documented that deltoid fatty infiltration often occurs after shoulder injury or denervation, but recent research has found other risk factors for fatty infiltration of the deltoid muscle. Rajabzadeh-Oghaz analyzed deltoid fatty infiltration via BONE kernel CT and FC30 kernel CT. These different CT modalities provide sharper images for specific anatomical applications. A kernel is a reconstruction algorithm applied to raw CT data to produce diagnostic images. The BONE kerne**l** enhances cortical bone detail and is optimized for high-resolution bone visualization. The FC30 kernel provides a smoother image suitable for soft tissue evaluation, though it still preserves sufficient bone detail. For both BONE kernel CT and FC30 kernel CT, men and women with greater fatty infiltration had significantly higher comorbidities.^21^ For men, a significantly greater percentage of fatty infiltration patients had diabetes.^21^ For women, a significantly greater percentage of fatty infiltration patients had inflammatory arthritis, hypertension, and diabetes.^21^

Additionally, Rajabzadeh-Oghaz et al^21^ found that both male and female patients with greater deltoid fatty infiltration had significantly higher body mass index (BMI). Matson et al^16^ analyzed the effect of obesity on fatty infiltration of the rotator cuff muscles as well as the three surrounding muscles (deltoid, trapezius, and teres major). It illustrated that obese patients tend to have greater levels of fatty infiltration. Using the GC system, they found that obese patients received a score of 0 less frequently than did non-obese patients (51% vs. 74%, *P* = .001).^16^ Also, distributions for Goutallier grades were significantly higher on average (*P* < .05) in larger BMI categories for 4 out of 6 of the muscles, including the deltoid muscle. A Goutallier grade of 2 or higher was assigned in 15.4% (45/293) of muscles of obese patients compared to 2.1% (6/281) of muscles for non-obese patients.^16^ The association between BMI and rotator cuff fatty infiltration may be explained by obesity-induced ectopic fat deposition in skeletal muscle. Past research explains several potential drivers of ectopic fat deposition, including reduced physical activity, chronic low-grade inflammation, and metabolic dysfunction.^23^^,^^26^

Comorbidities, such as diabetes, hypertension, and obesity, are associated with greater deltoid fatty infiltration, highlighting additional risk factors that orthopedic surgeons should consider prior to surgical intervention. Not only does it introduce other risk factors, but it also suggests how health disparities could impact outcomes following rTSA. Health disparities, driven by social determinants of health and higher prevalence of chronic diseases in certain racial and socioeconomic groups, may contribute to worse rTSA outcomes by increasing the likelihood of greater fatty infiltration. Future research should be conducted to determine how health disparities affect post-operative outcomes of rTSA. This would allow for awareness and potential solutions to mitigate health inequity.

Besides comorbidities, sex was also shown to be associated with differences in deltoid fatty infiltration. Sex was shown not to be significantly associated with fatty infiltration with the exception of the deltoid muscle, which showed a statistically significant increase in fatty infiltration associated with female sex (*P* = .038).^6^ Rajabzadeh-Oghaz et al^21^ also demonstrated that females had higher deltoid fat percentage than males, about 19%-23% vs. 18%-20% respectively. Future research should determine if greater deltoid fatty infiltration among women affects surgical outcomes post-rTSA. If so, it would be beneficial to devise strategies to mitigate any discrepancy.

This study has several limitations. One limitation among most of the included studies is sample size. Most of the studies had sample sizes of less than 100 patients with many of them even having significantly less patients than that. The small sample sizes of many of the studies in the systematic review propose potential issues with the generalizability and applicability of the findings. Besides the small sample sizes, there was some heterogeneity among the primary outcomes of the studies. As outlined earlier, the outcomes were determined by clinical and functional assessments, like ASES, CS, SAS, and KSS. Therefore, each study did not use the same modality for assessment. Moreover, only retrospective studies were used which limits the ability to infer causality.

## Conclusion

This study highlighted the significance in using deltoid fatty infiltration as a predictor of post-operative patient outcomes. Thus, understanding risk factors associated with deltoid fatty infiltration and the impact deltoid fatty infiltration has on rTSA outcomes may necessitate change in presurgical protocol to optimize patient outcomes. Knowing the degree of fatty infiltration, evaluated pre-operatively by MRI and CT imaging, can allow for a more individualized surgical approach. Compromised deltoid strength due to fatty infiltration can necessitate tailored surgical planning, including tuning the positioning of the glenoid and humeral component and choice of implant to optimize surgical outcomes. Rehabilitation strategies can also be implemented to accommodate and mitigate potential post-operative risks and complications due to fatty infiltration.

Focusing on fatty infiltration within the deltoid addresses an integral factor influencing rTSA post-operative success. Future studies can provide a more detailed examination of the relationship between fatty infiltration of the deltoid and rTSA. Leveraging advanced imaging techniques, mapping the localization of fatty infiltration of the deltoid, can provide a nuanced understanding of how distinct regions of fatty infiltration in the anterior, lateral, and posterior segments influence post-operative rTSA outcomes. Current assessments often rely on observational data and functional scoring systems; in the future, using MRI to quantify and map fatty infiltration throughout the deltoid can not only identify how specific regions might differentially contribute to rTSA outcomes but may also suggest pre-operative interventions to optimize surgical outcomes. Incorporating machine learning into imaging analyses can also enable identification of patterns in fatty infiltration and their correlations to rTSA outcomes. By combining these technologies, future research can refine the current understanding of fatty infiltration of the deltoid, illuminate details regarding rTSA outcomes, and optimize patient-specific treatment plans.

## Disclaimers

Funding: No funding was disclosed by the authors.

Conflicts of interest: The authors, their immediate family, and any research foundation with which they are affiliated have not received any financial payments or other benefits from any commercial entity related to the subject of this article.
